# Divergent effects of transformational leadership on safety compliance: A dual-path moderated mediation model

**DOI:** 10.1371/journal.pone.0262394

**Published:** 2022-01-24

**Authors:** Ting Wu, Yi Wang, Rebecca Ruan, Jianzhuang Zheng

**Affiliations:** 1 School of Business, Zhejiang University City College, Hangzhou, China; 2 Department of Regional Development Research, Zhejiang Development & Planning Institute, Hangzhou, China; 3 School of Geoscience, The University of Edinburgh, Edinburgh, Scotland, United Kingdom; American University of Sharjah, UNITED ARAB EMIRATES

## Abstract

Research on the relationship between transformational leadership and safety compliance has yielded equivocal results. This study investigates how and when transformational leadership produces divergent effects on safety compliance. Using a time-lagged research design, we collect data from a sample of 309 employees in the Chinese construction industry to examine the hypothesized relationship. We find that transformational leadership positively affects safety compliance through employees’ felt obligation toward their leader. However, transformational leadership also negatively impacts safety compliance through safety risk tolerance. We further show that employees’ perception of the safety climate plays a contingent role in the above processes. Specifically, a high-level perceived safety climate strengthens the positive indirect effect of transformational leadership on safety compliance through felt obligation, while a low-level perceived safety climate strengthens the negative indirect effect of transformational leadership on safety compliance through safety risk tolerance. The theoretical and practical implications of the findings are also discussed.

## Introduction

Workplace safety is a pressing concern for both researchers and practitioners in the field of safety management. Work-related injuries result in employee suffering and high organizational costs [1–3]. According to the U.S. Bureau of Labor Statistics survey, approximately 2.7 million nonfatal occupational injuries and illnesses were reported by private industry employers in the United States in 2020 [4]. In China, although the general data on similar workplace safety incidents are not publicly available; nonetheless, an increasing number of safety accidents—especially those that occur in the construction industry—are reported by the government and the media. For instance, a report from the Ministry of Emergency Management described the collapse of a plant that occurred on May 16, 2019 in Changning district, Shanghai and caused 12 deaths, 10 serious injuries, three minor injuries, and direct economic losses of approximately 34.3 million yuan [5]. As highlighted by Jiang and Xu (2020), safety accidents in the construction industry in China occur frequently and continue to increase, with 734 accidents and 840 fatalities reported in 2018 alone [6].

Given the importance of workplace safety, a growing body of research has investigated the antecedents of safety compliance (i.e., a form of in-role safety performance that refers to mandated safety behaviors that are usually part of the formal requirements of employees’ work roles) [7], such as employee personality [e.g., 8, 9], safety climate [e.g., 10, 11], and supervisor helping behavior [e.g., 12]. Transformational leadership is another significant antecedent of safety compliance that has been widely explored in research on workplace safety in recent years [e.g., 8, 13–16]. However, the body of research in this area has yielded equivocal results on the relationship between transformational leadership and safety compliance. Most studies have documented a positive impact of transformational leadership on safety compliance [e.g., 8, 13, 14]. However, some studies have found a non-significant association between these two variables [e.g., 15, 17]. In a study by Inness et al. (2010) across different job sectors, employees’ perceptions of transformational leadership were found not related to their safety compliance in either primary or secondary jobs [15]. The researchers suggested that higher levels of transformational leadership may indirectly give employees more freedom in deciding whether to abide by existing organizational policies such as safety procedures, which conversely lead to variability in safety compliance.

Moreover, some studies in the field of safety management have even suggested that transformational leadership may negatively affect safety compliance. In viewing transformational leadership as a complex and multidimensional construct, Hoffmeister et al. (2014) examined the distinct effects of different dimensions of transformational leadership on safety compliance, and found one of these—individualized consideration—to be negatively related to safety compliance [16]. A similar relationship was reported in a recent study conducted by Xue, Fan, and Xie (2020), who found that senior managers’ safety inspiration (another dimension of transformational leadership) is negatively associated with safety compliance [18]. While these mixed findings have attracted substantial research interest, the relationship between transformational leadership and safety compliance remains unclear. Clarke (2013) argued that the mixed findings in the literature may be due to past studies’ failure to account for the negative role of transformational leadership and the complicated process through which transformational leadership influences safety compliance [19].

To address this research gap, we use leader-member exchange (LMX) theory and self-regulatory focus (SRF) theory to explore the divergent effects of transformational leadership on safety compliance. Specifically, we investigate the two distinct pathways through which transformational leadership may influence safety compliance. According to LMX theory [20–22], transformational leadership can build high-quality exchange relationships and trigger their employees’ perceptions of felt obligation toward their leader, which in turn, encourage employees to adhere to safety regulations. According to SRF theory [23, 24], transformational leadership in the area of safety management can provide employees with autonomy in decision-making and foster their greater tolerance for risk-taking behaviors, thereby decreasing safety compliance. This study primarily addresses two aspects of the above theoretical predictions. First, we investigate two divergent paths by which transformational leadership may affect safety compliance: “transformational leadership—felt obligation to leaders—safety compliance” and “transformational leadership—safety risk tolerance—safety compliance.”. Second, we evaluate the conditional role of the perceived safety climate in determining the above relationships between transformational leadership and safety compliance.

**Fig 1:**
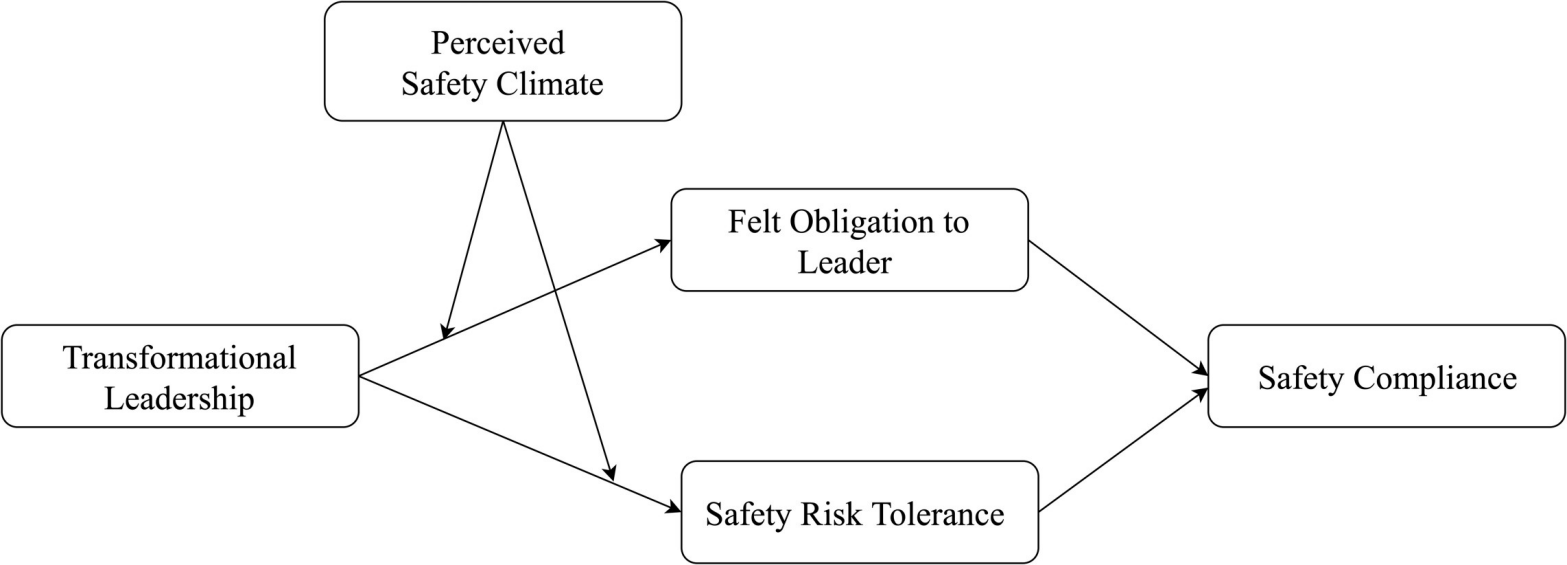
The dual-path model of transformational leadership on safety compliance.

The overarching research model is illustrated in Fig 1.

## Literature

### Transformational leadership, felt obligation to leader and safety compliance

LMX theory focuses on the quality of dyadic exchanges between leaders and followers. It holds that when it is difficult to allocate resources evenly due to limited resources and time pressure, leaders develop high-quality LMX relationships with in-group members that are upheld by mutual trust, reciprocal influence, loyalty, and a sense of mutual obligation [25]. In contrast, employees in low-quality LMX relationships that are shaped by their formal roles and organizational employment are unlikely to receive extra support from their leaders.

We expect transformational leadership to serve as a contextual element against which a sense of obligation to the leader may develop. Transformational leaders can provide employees with both the economic and social benefits that they expect through intellectual stimulation (which encourages employees to step out of their rigid mindset), the individualized consideration to satisfy their personalized needs, an idealized influence to promote trust and respect, and the inspirational motivation to trigger intrinsic motivation [26–28]. LMX theory contends that employees reciprocate favorable treatment by accepting the organizational goals set by their leaders, and increasingly adopt organization-oriented identities rather than self-centered ones [29, 30]. In addition, LMX theory holds that the dyadic relationship between leaders and employees is dynamic; that is, transformational leaders may encourage out-group elements to become in-group members, causing employees to feel a sense of obligation toward their leader. Several studies have shown empirically that employees are more likely to have a salient felt obligation toward their leader or organization when they perceive a high level of transformational leadership [e.g., 31, 32]. Hence, we propose the following hypothesis:

**H1a:** Transformational leadership has a positive effect on employees’ felt obligation to leaders.

Employees who feel a high level of obligation toward their leaders tend to adopt their leaders’ perspectives, be more likely to engage in prosocial organizational behaviors, and aim to achieve the organizational goals established by their leaders [33]. As a form of in-role safety performance, safety compliance refers to mandated safety behaviors that are usually part of the formal requirements of employees’ work roles [7]. In managing workplace safety, the leaders of organizations optimize safety rules or regulations to avoid high-cost work-related injuries. In line with this expectation, employees with a high level of felt obligation toward their leader tend to abide by safety goals. Thus, they demonstrate higher safety compliance. However, employees that lack feelings of felt obligation toward their leader tend to show lower safety compliance. Empirical studies have found that employees’ felt obligation to leaders is positively related to safety compliance [31, 34].

Overall, drawing on LMX theory, we infer that transformational leadership positively affects safety compliance by enhancing employees’ felt obligation to leaders. Transformational leaders are more likely to deliver economic and social benefits to their employees. By acting as role models and providing inspirational motivation, intellectual stimulation, and individualized consideration, transformational leaders create high-quality social exchange relationships with their employees. Such relationships enhance employees’ felt obligation to their leaders. In turn, employees with higher levels of felt obligation to leaders strive to meet their leaders’ expectations regarding safety behaviors and, consequently, demonstrate greater safety compliance. Hence, we propose the following hypotheses:

**H1b:** Employees’ felt obligation to leaders is positively associated with safety compliance.
**H1c:** Employees’ felt obligation to leaders mediates the relationship between transformational leadership and safety compliance.

### Transformational leadership, safety risk tolerance, and safety compliance

SRF theory, proposed by Higgins (1998) [24], describes two distinct self-regulatory states that are involved in individual decision-making: promotion focus and prevention focus. Individuals who demonstrate promotion focus are more eager to attain advancement, growth, and accomplishment. In contrast, individuals who demonstrate prevention focus are more concerned with goals that are related to their duties, obligations, and necessities. Given these differences, we expect individuals to behave differently in safety management according to whether they display a stronger promotion focus or prevention focus.

Studies have shown that leaders often suppress their employees’ prevention focus by exhibiting transformational leadership [7, 17], such as by providing inspirational motivation and intellectual stimulation. When leaders motivate their employees (e.g., by portraying a better future for the organization or the forward direction of the struggle), those employees become more inclined to pursue positive results (e.g., advancement, growth, and accomplishment) rather than avoid negative results (e.g., external threats, safety risks, and absence from duties). Similarly, through intellectual stimulation (e.g., by encouraging employees to think of solutions from various perspectives and of ways to resolve problems innovatively), leaders restrain their employees’ prevention focus while stimulating their promotion focus. In such circumstances, employees tend to be less sensitive to negative results, thus leading to higher safety risk tolerance. Hence, we propose the following hypothesis:

**H2a:** Transformational leadership has a positive effect on safety risk tolerance.

There is generally a lack of research investigating safety risk tolerance and the subsequent safety decisions regarding the safety behaviors [35, 36]. Nonetheless, a growing body of primary and secondary evidence suggests that employees with higher safety risk tolerance are more likely to demonstrate risk-taking behaviors, such as reduced safety compliance. According to Bhandari and Hallowell (2022), employees with higher safety risk tolerance underestimate the safety risks in their work routines or simply regard safety activities as being “part of the job.” Such normalized risk-taking attitudes cause workers to resent the safety measures imposed by management, ultimately resulting in compliance issues such as the inappropriate use of personal protective equipment [37]. Given the dynamic nature of construction work, safety-related behaviors are difficult to monitor, regulate, and control in real-time. Globally, the construction industry has struggled to reduce workers’ willingness to accept risks (i.e., to lower their safety risk tolerance) through various safety training programs [38, 39]. This serves to demonstrate the universal importance of safety risk tolerance in management practices on construction sites to prevent safety incidents. SRF theory holds that employees whose prevention focus is restrained and whose promotion focus is stimulated are more likely to be intrinsically motivated, overestimate their ability to control or prevent accidents, and ignore external threats [40]. This behavior leads to reduced risk identification, biased risk analysis, and inaccurate evaluations of the risks pertaining to safety accidents or injuries.

Drawing from the above arguments, we contend that safety risk tolerance mediates the relationship between transformational leadership and safety compliance. SRF theory contends that when leaders demonstrate higher transformational leadership—for instance, through inspirational motivation and intellectual stimulation—employees increase their acceptable amount of risks in the pursuit of safety goals, thus resulting in a lack of risk assessments and less safety compliance. Hence, we propose the following hypotheses:

**H2b:** Safety risk tolerance is negatively related to safety compliance.
**H2c:** Safety risk tolerance mediates the relationship between transformational leadership and safety compliance.

### Conditional role of the perceived safety climate

As discussed, transformational leadership influences safety compliance through two pathways. Nonetheless, both pathways are influenced by the organizational context [3, 41, 42]. At the individual level, the safety climate is a crucial and pervasive organizational context that shapes safety behaviors and outcomes [43–45]. Zohar (2010) defined the safety climate as the individual’s perceptions of safety policies, procedures, and practices in an organization. Empirical studies across a range of industries have demonstrated the influence of the safety climate on safety behaviors [2, 43, 46]. Therefore, in this study, we consider the safety climate to be a moderator and propose an integrated hypothesis: the indirect association between transformational leadership and safety compliance through employees’ felt obligation to leaders (safety risk tolerance) is conditionally dependent upon their perceptions of the safety climate.

Specifically, when employees perceive a high-level safety climate, transformational leadership is more likely to influence their safety compliance via their felt obligation to leaders. The safety climate reflects the extent to which the management prioritizes general safety policies, formal safety procedure systems, and safety practices [47]. Employees who perceive a high-level safety climate are likely to conscious of these priorities, as well as the corresponding behaviors that may be rewarded or sanctioned. This consciousness allows them to gain a deeper understanding of the complex relationships that exist between competing organizational goals (e.g., productivity vs. safety), time frames (e.g., short- vs. long-term goals), and contradictory messages (e.g., enacted vs. declared policies) [48]. Therefore, when adhering to safe work practices, employees who perceive a strong safety climate focus on the behaviors of leaders, as well as their relevance or value to the organization. This occurs because the safety climate usually reflects leaders’ expectations, concerns, and requirements regarding safety.

However, when employees perceive a low-level safety climate, the alternative pathway—in which transformational leadership influences safety compliance via safety risk tolerance—is more likely to occur. In such circumstances, employees’ prevention focus is further inhibited, and they are less sensitive to potential safety risks, hazards, or accidents in their daily work activities [49, 50], because they assume that their leaders place less importance on safety goals. Hence, employees’ safety risk tolerance increases when facing a low-level perceived safety climate, resulting in lower safety compliance. We therefore propose the following hypotheses:

**H3a:** The indirect effect of transformational leadership on safety compliance through employees’ felt obligation to leaders is conditional on the perceived safety climate, such that a higher-level perceived safety climate strengthens this indirect effect.
**H3b:** The indirect effect of transformational leadership on safety compliance through employees’ safety risk tolerance is conditional on the perceived safety climate, such that a lower-level perceived safety climate strengthens this indirect effect.

## Materials and methods

### Sample and procedures

The study was approved by the Academic Committee of Zhejiang University City College. All participants provided written informed consent when they were filling out the questionnaires. The study addressed a large Chinese construction company employing over 5000 construction workers. Several safety issues concerned the company’s staff, including falling hazards, electric accidents, and unsafe environments. We conducted two rounds of questionnaire collection. Before the workers filled out the questionnaires in each round, we asked them to take two minutes to read the introduction of the study, in which we guaranteed them that all data would be limited to scientific research and would not be presented to their supervisors or employers. To further enhance the anonymity of the data, we asked the workers to fill in the last six digits of their phone numbers instead of their names. By doing so, we were able to match the two rounds of questionnaires based on the phone numbers, while mitigating the concerns of data leakage among the construction workers.

We also used a time-lagged data collection to reduce common method bias [51],. At Time 1, the construction workers provided measures for transformational leadership, felt obligation to leaders, safety risk tolerance, and control variables. At Time 2 (two months after the survey), the construction workers were asked to rate safety compliance and perceived safety climate. In total, we received 420 questionnaires at Time 1 (84% response rate) and 348 questionnaires at Time 2 (73% response rate). The final matched and valid sample consisted of 309 construction workers with an average age of 39.2 years. Due to the nature of the high-intensity work in the construction industry, the study’s sample predominantly comprised men (88% were male) and was characterized by low education (primary education at 26.9%, secondary education at 59.2%).

### Measurement

All the above measures were administered in Chinese and translated from English into Chinese by using a procedure of standard translation and back-translation to ensure validity [52]. At the initial translation stage, two experts with a high level of English, one being the author of our research and the other being a manager from the construction industry, translated all the questionnaire items from English to Chinese. They then proceeded to check, discuss, and modify the translated Chinese questionnaire items until both agreed on the results of all the items. After that, in the back-translation stage, we paid an English graduate student to re-translate the Chinese questionnaire items obtained in the previous stage into English and compare them with the original English questionnaire items. If the meanings expressed do not match, the translation phase is repeated until they agree on the meaning. Items regarding the felt safety climate were measured on a seven-point rating scale ranging from “1” (strongly disagree) to “7” (strongly agree), while the remaining variables were measured on five-point scales ranging from “1” (strongly disagree) to “5” (strongly agree).

#### Transformational leadership

Leadership was measured using 20 items from the Multifactor Leadership Questionnaire (MLQ) [53]. The MLQ measures the components of transformational leadership, such as idealized influence, inspirational motivation, intellectual stimulation, and individualized consideration. Employees reported how they feel about their leader behaviors in these aspects. Sample items include: “My supervisor acts in a way that builds my respect,” “My supervisor talks optimistically about the future to me,” “My supervisor seeks differing perspectives from me when solving problems,” and “My supervisor spends time teaching and coaching me.” The alpha coefficient of the scale is.94.

#### Felt obligation to leader

To measure an employee’s desire to repay his/her leader, we adapted Liang et al.’s (2012) five-item felt-obligation scale and changed the referent from organization to leader [54]. An example item was: “I feel a personal obligation to do whatever I can to help my leader achieve his/her goals.” The alpha coefficient of the scale is.89.

#### Safety risk tolerance

We used Wang et al.’s (2016) eight-item scale of safety risk tolerance, which covers the most common construction accidents and injuries (falling hazard, electric accident, and unsafe environment, among the others), to measure how seriously construction workers perceived the confronted risks [55]. Three items were later discarded owing to low factor loadings. An example item was: “I can accept that safety nets do not cover the building when construction is in progress.” The alpha coefficient of the scale is .82.

#### Felt safety climate

We used Neal and Griffin’s (2006) three-item safety climate scale to measure the degree to which safety was valued by the organization [56]. Sample items include the following: “Management places a strong emphasis on workplace health and safety” and “Safety is given a high priority by management.” The alpha coefficient of the scale is.86.

#### Safety compliance

A three-item scale by Neal and Griffin (2006) was used to operationalize safety compliance in terms of safety activities that should be carried out by construction workers to maintain workplace safety. Sample items include the following: “I use all the necessary safety equipment to do my job” and “I ensure the highest levels of safety when I carry out my job.” The alpha coefficient of the scale is.88.

#### Control variables

We also controlled for age, gender, and education level, as a review of previous research suggests that these employees’ demographic variables may significantly impact safety compliance [7, 42].

### Method of analysis

In this study, we adopted software SPSS 25.0, SPSS AMOS, and MPLUS 7.0 as data analysis tools to analyze the 309 valid data. The data analysis approaches involved in this research include (1) confirmatory factor analysis (CFA), (2) tests of convergent and discriminant validity, (3) descriptive statistics and correlations, and (4) hypotheses testing.

We ran a CFA using SPSS AMOS to check whether the hypothesized five-factor model matched the actual data. We used five general indexes to evaluate the model fit: CFI, IFI, TLI, RMSEA, and SRMR. Additionally, we conducted tests of convergent and discriminant validity for the hypothesized variables. Specifically, we used the indicators of average variance extracted (AVE) and composite reliability (CR) to verify the convergent validity and the comparisons of AVE with the squared correlations involving the variables (AVE/*r*^*2*^) to verify the discriminant validity [57].

While testing the direct effects among variables (Hypotheses 1a, 1b, 2a, and 2b), we conducted hierarchical linear regression in SPSS 25.0. In the process of analysis, Model 1 includes the control variables and the dependent variable of safety compliance; Model 2 includes the control variables, the independent variable of transformational leadership, and the dependent variable of safety compliance; Model 3 includes the control variables, the independent variable of transformational leadership, and the dependent variable of felt obligation to leader; Model 4 includes the control variables, the independent variable of transformational leadership, and the dependent variable of safety risk tolerance; Model 5 includes the control variables, the independent variable of felt obligation to leader, and the dependent variable of safety compliance; Model 6 includes the control variables, the independent variable of safety risk tolerance, and the dependent variable of safety compliance.

To test the hypothesized mediation effects, we used a bootstrap method, applying MPLUS 7.0. We used 5000-sample bootstrapping to yield 95% bias-corrected confidence intervals. If the confidence interval excludes zero, it leads to the inference that the mediation effect is significant. In the final step, we then used MPLUS 7.0 to quantify the difference in mediation effects between low (-1SD) and high (+1SD) levels of perceived safety climate. If the confidence interval of the difference excludes zero, it leads to the inference that the moderating effect is significant.

## Results

### Confirmatory factor analysis

To provide evidence of construct distinctness, we used the software AMOS to conduct CFA on the survey items addressing five variables: transformational leadership, felt obligation to leaders, safety risk tolerance, perceived safety climate, and safety compliance. Using data obtained from 309 questionnaires, we compared five alternative models with the baseline model, five-factor Model 1. As shown in Table 1, the hypothesized five-factor structure of Model 1, with all items loading on their respective factors, fit the data in an acceptable manner, with χ^2^ [142, n = 309] = 333.51, RMSEA = 0.066, CFI = 0.94, IFI = 0.94, TLI = .93, and SRMR = 0.060 [58]. The proposed model guarantees a substantial improvement in fit indexes compared to alternative models (Models 2–6). In addition, all standardized factor loadings were above .40 and significant. These results suggest that the five constructs captured distinctiveness as expected.

**Table 1:** Comparison of measurement models.

Models	Factors	*χ* ^*2*^	*df*	*Δχ* ^*2*^	RMSEA	CFI	IFI	TLI	SRMR
1	*Five factors*: Transformational leadership, felt obligation to leader, safety risk tolerance, perceived safety climate, safety compliance	333.51	142		.066	.94	.94	.93	.060
2	*Four factors*: Transformational leadership and perceived safety climate combined into one factor.	749.92	146	416.41**	.115	.82	.82	.79	.096
3	*Four factors*: Felt obligation to leader and safety risk tolerance combined into one factor.	814.44	146	480.93**	.122	.80	.80	.77	.116
4	Three factors: Felt obligation to leader, safety risk tolerance and perceived safety climate combined into one factor.	1405.98	149	1072.47**	.165	.62	.63	.57	.173
5	*Two factors*: Time 1 variables (e.g., transformational leadership) combined into one factor; Time 2 variables (e.g., safety compliance) combined into one factor.	2165.18	151	1831.67**	.208	.40	.40	.32	.209
6	*Single factor*	2525.31	152	2191.80**	.225	.29	.29	.20	.220

### Tests of convergent and discriminant validity

According to Ahmad et al. (2016), AVE and CR can be used to assess the convergent validity, while AVE/*r*^*2*^ can be used to test the discriminant validity [57]. As shown in Table 2, all the CR values are above 0.70, and the AVE values are above 0.5, indicating good convergent validity for all variables. Moreover, the results of discriminant validity tests (AVE/*r*^*2*^ > 1) for all variables suggest that the amount of the variance capture by each variable is greater than the shared variance with the other variables, indicating that the variables are distinct from one another.

**Table 2:** Tests for convergent and discriminant validity.

Variables	Convergent validity	Discriminant validity
Transformational leadership		
CR	0.906	AVE/*r*^*2*^ > 1
AVE	0.706	
Felt obligation to leader		
CR	0.883	AVE/*r*^*2*^ > 1
AVE	0.604	
Safety risk tolerance		
CR	0.825	AVE/*r*^2^ > 1
AVE	0.556	
Perceived safety climate,		
CR	0.885	AVE/*r*^*2*^ > 1
AVE	0.721	
Safety compliance		
CR	0.864	AVE/*r*^*2*^ > 1
AVE	0.697	

### Descriptive statistics

Table 3 presents the descriptive statistics, reliabilities, and correlations for the proposed variables. As expected, transformational leadership was found to be positively related to the felt obligation to leaders (*r* = .18, *p* < .01) and safety risk tolerance (*r* = .18, *p* < .01). Furthermore, these correlation results also reveal that felt obligation to leaders has a positive relationship with safety compliance (*r* = .34, *p* < .01), while safety risk tolerance has a negative relationship with safety compliance (*r* = −.12, *p* < .05).

**Table 3:** Descriptive statistics, reliabilities, and inter-correlations among the hypothesized variables.

Variables	M	SD	1	2	3	4	5	6	7	8
1. Age	39.18	8.80	—							
2. Gender	1.12	.33	−.07	—						
3. Education	1.87	.63	−.29**	.01	—					
4. Transformational leadership	3.85	.66	−.20**	.00	.08	(.94)				
5. Felt obligation to leader	3.36	1.03	−.09	.09	.05	.18**	(.89)			
6. Safety risk tolerance	3.48	.81	.01	−.05	.12*	.18**	.00	(.82)		
7. Felt safety climate	5.42	1.21	−.11	−.05	.13*	.45**	−.11	.21**	(.86)	
8. Safety compliance	4.08	.79	.01	.08	−.06	.12*	.34**	−.12*	−.03	(.88)

*Note*: *N* = 309.

* *p* < .05

** *p* < .01. Internal consistency reliabilities are reported in parentheses along diagonal.

### Hypothesis testing

Table 4 presents the results of hierarchical regression analyses for the Hypotheses 1a, 1b, 2a, and 2b. As predicted, controlling for the above demographic variables, we found that transformational leadership is positively related to felt obligation to leaders (β = .26, p < .01, ΔR^2^ = 0.03), and transformational leadership accounted for 3% of the variance in felt obligation to leaders. Similarly, we found that transformational leadership is positively related to safety risk tolerance (β = .22, p < .01, ΔR^2^ = 0.04), and transformational leadership accounted for 4% of the variance in safety risk tolerance. We also found that felt obligation to leaders is positively related to safety compliance (β = .27, p < .01, ΔR^2^ = 0.12), whereas safety risk tolerance is negatively related to safety compliance (β = −.11, p < .05, ΔR^2^ = 0.01), suggesting that felt obligation to leaders and safety risk tolerance accounted for 12% and 1% of the variance of safety compliance, respectively. Overall, these results support Hypotheses 1a, 1b, 2a, and 2b.

**Table 4:** Results of hierarchical regression analyses.

	Model 1	Model 2	Model 3	Model 4	Model 5	Model 6
	DV: SC	DV: SC	DV: FOL	DV: SRT	DV: SC	DV: SC
**Age**	–.00 (.01)	.00 (.01)	–.01 (.01)	.01 (.01)	.00 (.01)	.00 (.01)
**Gender**	–.19 (.14)	.19 (.14)	.26 (.18)	–.11 (.14)	.12 (.13)	.18 (.14)
**Education**	–.08 (.07)	–.08 (.07)	.04 (.10)	.16* (.07)	–.09 (.07)	–.06 (.07)
**Transformational leadership (TL)**		.15* (.07)	.26** (.09)	.22** (.07)		
**Felt obligation to leader (FOL)**					.27** (.04)	
**Safety risk tolerance (SRT)**						–.11* (.06)
***R* ^*2*^**	.01	.03	.04	.05	.13	.02
***ΔR* ^*2*^**		.02	.03	.04	.12	.01

*Note*. *N* = 309.

* *p* < .05

** *p* < .01. The standard errors in the estimations are reported in parentheses. TL is for transformational leadership. FOL is for felt obligation to leader, SRT is for safety risk tolerance. SC is for safety compliance. Model 1 is the base model for Model 2–6.

To verify the mediating role of felt obligation and risk tolerance, we adopted bootstrap methods to test their indirect effects by using the software MPLUS 7.0 [59]. The results are reported in Table 5 and show that the indirect effect of transformational leadership on safety compliance via felt obligation to leaders is equal to 0.07, with 95% confidence interval (CI) = [.031,.127], and the indirect effect of transformational leadership on safety compliance via safety risk tolerance is −0.03, with 95% CI = [−.079, −.004]. Since all confidence intervals exclude zero, Hypothesis 1c and 2c are supported.

**Table 5:** Mediation effects of felt obligation to leader and safety risk tolerance.

The Dual Paths	Mediation effect	95% CI of indirect effect, 5000 bootstrap sampling
TL→ FOL → SC	.07**	CI = [.031, .127]
TL→ SRT → SC	−.03*	CI = [−.079, −.004]

*Note*: *N* = 309.

* *p* < .05

** *p* < .01. TL is for transformational leadership. FOL is for felt obligation to leader, SRT is for safety risk tolerance. SC is for safety compliance.

Hypotheses 3a and 3b contended that a high-level perceived safety climate enhances the positive indirect effect of transformational leadership affecting safety compliance through the felt obligation to leaders, while a low-level perceived safety climate enhances the negative indirect effect of transformational leadership affecting safety compliance through safety risk tolerance. To gain an explicit insight into how the indirect effects differ depending on the values of the perceived safety climate, we employed a bootstrapping procedure to quantify the indirect effects at low (-1SD) and high (+1SD) levels of perceived safety climate by using the software MPLUS 7.0. Table 6 reports the indirect effects at different values of perceived safety climate and provides the 95% CI for these effects. In line with Hypothesis 3a, the positive indirect effect in Path 1 (Transformational leadership–felt obligation to leaders–safety compliance) is stronger at high (γ = .16, 95% CI = [.090,.255]) than low ((γ = .07, 95% CI = [.012,.163]) levels of perceived safety climate. An additional test showed that the difference between these two indirect effects is statistically significant (Δγ = .09, CI = [.004,.189]). Similarly, we can observe that, in line with Hypothesis 3b, the negative indirect effect in Path 2 (Transformational leadership–safety risk tolerance–safety compliance) is stronger at low (γ = −.05, 95% CI = [−.108, −.004]) than high ((γ = .02, 95% CI = [−.004,.065]) levels of perceived safety climate. An additional test confirmed that the difference between these two indirect effects is statistically significant (Δγ = .07, CI = [.005,.149]). Therefore, Hypotheses 3a and 3b are both supported.

**Table 6:** Conditional indirect effects of transformational leadership on safety compliance at values of perceived safety climate.

Moderator	Effect	Moderated mediation effect	95% CI of moderated mediation effect
Path 1: TL→ FOL → SC			
Low felt safety climate (−1 SD)	.26**	.07*	[.012, .163]
High felt safety climate (+1 SD)		.16**	[.090, .255]
Differ		.09*	[.004, .189]
Path 2: TL→ SRT → SC			
Low felt safety climate (−1 SD)	−.120*	−.05*	[−.108, −.004]
High felt safety climate (+1 SD)		. 02	[−.004, .065]
Differ		.07*	[.005, .149]

*Note*. *N* = 309.

* *p* < .05

** *p* < .01. TL is for transformational leadership. FOL is for felt obligation to leader, SRT is for safety risk tolerance. SC is for safety compliance.

## Discussion

Guided by LMX and SRF theory, we examined how transformational leadership exerts divergent effects on safety compliance using time-lagged data from the Chinese construction industry. The study’s main findings are as follows. First, transformational leadership has a positive effect on safety compliance through the felt obligation to leaders, and simultaneously, a negative effect on safety compliance through safety risk tolerance. Second, a high-level perceived safety climate strengthens the above positive indirect effect, whereas a low-level perceived safety climate strengthens the above negative indirect effect. These findings contribute to the existing safety compliance literature and have important implications for safety practices within organizations.

### Theoretical and practical contributions

This study’s primary theoretical contribution lies in its novel integration of the LMX and SRF theories to yield a nuanced picture of the relationship between transformational leadership and safety compliance in workplace safety management. Studies on safety compliance have long observed the positive effects of transformational leadership, which fosters employees’ intrinsic motivations [60], feelings of trust [61], and greater sensitivity to job-related characteristics [62]. Previous studies have also suggested a negative or non-significant effect of transformational leadership on safety compliance [7, 19]. However, the equivocal relationship between transformational leadership and safety compliance has largely been overlooked and requires further investigation. In line with the “double-edged sword” effect of transformational leadership [63, 64], we propose a dual-path model of the effect of transformational leadership on safety compliance, which considers both employees’ felt obligation to leaders and safety risk tolerance based on the LMX and SRF theories, respectively. We found evidence for both the positive and negative effects of transformational leadership on safety compliance coexist. Thus, we offer a possible explanation for the mixed results across various studies. Specifically, we contend that a positive impact is observed when the effect of transformational leadership on safety compliance through the felt obligation to leaders exceeds the negative effect of transformational leadership on safety compliance through safety risk tolerance. In the opposite scenario, a negative effect would be observed. In some cases, these two effects, which are produced by different pathways, may offset each other, thus resulting in a non-significant impact of transformational leadership on safety compliance.

The study’s second key theoretical contribution is the use of the perceived safety climate to further investigate the mechanisms through which transformational leadership affects safety compliance [46]. We found that employees’ felt safety climate can increase their safety compliance, even when transformational leadership plays a negative role in safety management. This result confirms the significance of the safety climate in motivating employees to behave safely, even when their safety risk tolerance levels are incompatible with the organization’s safety goals. Furthermore, by considering the role of perceived safety climate on two alternative pathways, we also find that the perceived safety climate can serve as a favorable context. This context strengthens the positive effect of the pathway mediated by employees’ felt obligation to leaders and attenuates the negative effects of the pathway mediated by safety risk tolerance. This finding further clarifies that transformational leadership influence employees’ safety compliance under different conditions.

Although companies aim for their employees to guarantee high standards of safety compliance, transformational leadership may actually have a negative impact on safety compliance. Thus, leaders should be aware of the “double-edged sword” effect of transformational leadership on safety compliance. Leaders should use this knowledge strategically to facilitate their employees’ felt obligation toward them, especially employees with high safety risk tolerance. In addition, to maximize the positive impact of transformational leadership on employees’ safety compliance, companies should remind employees that safety in the workplace is a priority. For example, to enhance their employees’ perceptions of the organizational safety climate, companies should regularly communicate information regarding safety policies to their employees, involve them in the optimization of safety procedures, or reward them for their extraordinary achievements in safety practices.

### Limitations and future research

Several potential limitations of the current study should be noted. First, although we conducted the investigation at the individual level, this approach may have reduced the sensitivity of the proposed measures in comparison with differences in leadership and climate at the team or organizational levels. Previous studies have identified the cross-level impacts of transformational leadership and safety climate on safety compliance [e.g., 65, 66]. Thus, it would be interesting to explore the extent to which the results of the current remain valid when transformational leadership or safety climate are positioned at a higher level.

Second, the generalizability of the results may be limited owing to the distinct characteristics of the study sample. Although data from the construction industry allowed for exploring safety-related issues, it provided a biased sample in terms of gender and education. For instance, the study sample was

characterized by a particularly low percentage of female workers (less than 15%) and a relatively high percentage of workers with an education level lower than high school (more than 75%). Hence, the results of this study on data obtained from exclusively targeting the construction industry should be generalized with caution. Additional research based on balanced gender, education, and industry data is needed for validating the study results.

The third limitation of our study concerns the use of the transformational leadership scale in the MLQ. While MLQ is still widely recognized by the academic community, a recent criticism on the validity of its constructs suggested that the factorial structure of transformational leadership in MLQ should be classified formative (a latent model), rather than reflective (an aggregated model) [67]. The model misspecification of transformational leadership may result in erroneous parameter estimates and misleading statistical tests. This idea is supported to some extent when we elaborated on the possible negative relationship between transformational leadership and safety compliance. For instance, Hoffmeister et al. (2014) examined the distinct effects of different dimensions of transformational leadership on safety compliance and found one of these—individualized consideration—to be negatively related to safety compliance [16]. Therefore, future research could open the "black box" of transformational leadership to explore the distinct pathways of specific dimensions of transformational leadership affecting safety compliance. This will add explanations for the fundamental understanding of our research question: why has research on the relationship between transformational leadership and safety compliance yielded equivocal results?

## Conclusion

This study makes significant contributions to the safety compliance literature by examining how and when transformational leadership influences safety compliance. Using time-lagged data, we found that transformational leadership has a positive impact on safety compliance through the felt obligation to leaders and a negative impact on safety compliance through safety risk tolerance. We also introduced the perceived safety climate as an organizational context and found that a high-level perceived safety climate plays a favorable contingent role in achieving safety compliance. Thus, we concluded that transformational leadership is a “double-edged sword” in safety management, and employees’ perceptions of safety climate play a significant role in resolving the conflicts derived from the double-edged sword effect of transformational leadership on safety compliance. Hence, this study’s findings may be of significant help for organizations to develop effective safety interventions and practices.

## Supporting information

S1 Table Comparison of measurement models in the main study. (DOCX)

S2 Table Tests for convergent and discriminant validity. (DOCX)

S3 Table Descriptive statistics, reliabilities, and inter-correlations among the hypothesized variables. (DOCX)

S4 Table Results of hierarchical regression analyses. (DOCX)

S5 Table Mediation effects of felt obligation to leader and safety risk tolerance. (DOCX)

S6 Table Conditional indirect effects of transformational leadership on safety compliance at values of perceived safety climate. (DOCX)

S1 Questionnaire Questionnaire items (English and Chinese versions). (DOCX)

S1 Data (SAV)
