# Supplementary material for: Divergent effects of transformational leadership on safety compliance: A dual-path moderated mediation model
Source: PLoS One. 2022 Jan 24;17(1):e0262394. doi: 10.1371/journal.pone.0262394 (PMC8786187; doi:10.1371/journal.pone.0262394)
Supplement: S1 Table — (DOCX) [file pone.0262394.s001.docx]

**Table 1.** Comparison of Measurement Models

| **Models** | **Factors** | ***χ^2^*** | ***df*** | ***Δχ^2^*** | **RMSEA** | **CFI** | **IFI** | **TLI** | **SRMR** |
| --- | --- | --- | --- | --- | --- | --- | --- | --- | --- |
| 1 | *Five factors:* Transformational leadership, felt obligation to leader, safety risk tolerance, perceived safety climate, safety compliance | 333.51 | 142 |  | .066 | .94 | .94 | .93 | .060 |
| 2 | *Four factors:* Transformational leadership and perceived safety climate combined into one factor. | 749.92 | 146 | 416.41^**^ | .115 | .82 | .82 | .79 | .096 |
| 3 | *Four factors:* Felt obligation to leader and safety risk tolerance combined into one factor. | 814.44 | 146 | 480.93^**^ | .122 | .80 | .80 | .77 | .116 |
| 4 | Three factors: Felt obligation to leader, safety risk tolerance and perceived safety climate combined into one factor. | 1405.98 | 149 | 1072.47^**^ | .165 | .62 | .63 | .57 | .173 |
| 5 | *Two factors:* Time 1 variables (e.g., transformational leadership) combined into one factor; Time 2 variables (e.g., safety compliance) combined into one factor. | 2165.18 | 151 | 1831.67^**^ | .208 | .40 | .40 | .32 | .209 |
| 6 | *Single factor* | 2525.31 | 152 | 2191.80^**^ | .225 | .29 | .29 | .20 | .220 |
