# Supplementary material for: Divergent effects of transformational leadership on safety compliance: A dual-path moderated mediation model
Source: PLoS One. 2022 Jan 24;17(1):e0262394. doi: 10.1371/journal.pone.0262394 (PMC8786187; doi:10.1371/journal.pone.0262394)
Supplement: S2 Table — (DOCX) [file pone.0262394.s002.docx]

**Table 2.** Tests for Convergent and Discriminant Validity

| **Variables** | **Convergent validity** | **Discriminant validity** |
| --- | --- | --- |
| Transformational leadership |  |  |
| CR | 0.906 | AVE/*r^2^* > 1 |
| AVE | 0.706 |  |
| Felt obligation to leader |  |  |
| CR | 0.883 | AVE/*r^2^* > 1 |
| AVE | 0.604 |  |
| Safety risk tolerance |  |  |
| CR | 0.825 | AVE/*r*^2^ > 1 |
| AVE | 0.556 |  |
| Perceived safety climate, |  |  |
| CR | 0.885 | AVE/*r^2^* > 1 |
| AVE | 0.721 |  |
| Safety compliance |  |  |
| CR | 0.864 | AVE/*r^2^* > 1 |
| AVE | 0.697 |  |
