# Supplementary material for: Divergent effects of transformational leadership on safety compliance: A dual-path moderated mediation model
Source: PLoS One. 2022 Jan 24;17(1):e0262394. doi: 10.1371/journal.pone.0262394 (PMC8786187; doi:10.1371/journal.pone.0262394)
Supplement: S3 Table — (DOCX) [file pone.0262394.s003.docx]

**Table 3**. Descriptive statistics, reliabilities, and inter-correlations among the hypothesized variables

| **Variables** | **M** | **SD** | **1** | **2** | **3** | **4** | **5** | **6** | **7** | **8** |
| --- | --- | --- | --- | --- | --- | --- | --- | --- | --- | --- |
| 1. Age | 39.18 | 8.80 | — |  |  |  |  |  |  |  |
| 2. Gender | 1.12 | .33 | −.07 | — |  |  |  |  |  |  |
| 3. Education | 1.87 | .63 | −.29^**^ | .01 | — |  |  |  |  |  |
| 4. Transformational leadership | 3.85 | .66 | −.20^**^ | .00 | .08 | (.94) |  |  |  |  |
| 5. Felt obligation to leader | 3.36 | 1.03 | −.09 | .09 | .05 | .18^**^ | (.89) |  |  |  |
| 6. Safety risk tolerance | 3.48 | .81 | .01 | −.05 | .12^*^ | .18^**^ | .00 | (.82) |  |  |
| 7. Felt safety climate | 5.42 | 1.21 | −.11 | −.05 | .13^*^ | .45^**^ | −.11 | .21^**^ | (.86) |  |
| 8. Safety compliance | 4.08 | .79 | .01 | .08 | −.06 | .12^*^ | .34^**^ | −.12^*^ | −.03 | (.88) |

*Note: N*=309. ^*^ *p* < .05, ^**^ *p* < .01. Internal consistency reliabilities are reported in parentheses along diagonal.
