# Supplementary material for: Divergent effects of transformational leadership on safety compliance: A dual-path moderated mediation model
Source: PLoS One. 2022 Jan 24;17(1):e0262394. doi: 10.1371/journal.pone.0262394 (PMC8786187; doi:10.1371/journal.pone.0262394)
Supplement: S4 Table — (DOCX) [file pone.0262394.s004.docx]

**Table 4.** Results of hierarchical regression analyses.

|  | Model 1  DV: SC | Model 2  DV: SC | Model 3  DV: FOL | Model 4  DV: SRT | Model 5  DV: SC | Model 6  DV: SC |
| --- | --- | --- | --- | --- | --- | --- |
| Age | –.00 (.01) | .00 (.01) | –.01 (.01) | .01 (.01) | .00 (.01) | .00 (.01) |
| Gender | –.19 (.14) | .19 (.14) | .26 (.18) | –.11 (.14) | .12 (.13) | .18 (.14) |
| Education | –.08 (.07) | –.08 (.07) | .04 (.10) | .16* (.07) | –.09 (.07) | –.06 (.07) |
| Transformational leadership (TL) |  | .15* (.07) | .26** (.09) | .22** (.07) |  |  |
| Felt obligation to leader (FOL) |  |  |  |  | .27** (.04) |  |
| Safety risk tolerance (SRT) |  |  |  |  |  | –.11* (.06) |
| *R^2^* | .01 | .03 | .04 | .05 | .13 | .02 |
| *ΔR^2^* |  | .02 | .03 | .04 | .12 | .01 |

*Note. N*= 309. ^*^ *p* < .05, ^**^ *p* < .01. The standard errors in the estimations are reported in parentheses. TL is for transformational leadership. FOL is for felt obligation to leader, SRT is for safety risk tolerance. SC is for safety compliance. Model 1 is the base model for Model 2-6.
