# Supplementary material for: Divergent effects of transformational leadership on safety compliance: A dual-path moderated mediation model
Source: PLoS One. 2022 Jan 24;17(1):e0262394. doi: 10.1371/journal.pone.0262394 (PMC8786187; doi:10.1371/journal.pone.0262394)
Supplement: S5 Table — (DOCX) [file pone.0262394.s005.docx]

**Table 5.** Mediation effects of felt obligation to leader and safety risk tolerance.

| The Dual Paths | Mediation effect | 95% CI of indirect effect,  5000 bootstrap sampling |
| --- | --- | --- |
| TL→ FOL → SC | .07^**^ | CI= [.031, .127] |
| TL→ SRT → SC | −.03^*^ | CI= [−.079, −.004] |

*Note: N* = 309. ^*^ *p* < .05, ^**^ *p* < .01. TL is for transformational leadership. FOL is for felt obligation to leader, SRT is for safety risk tolerance. SC is for safety compliance.
