# Supplementary material for: Divergent effects of transformational leadership on safety compliance: A dual-path moderated mediation model
Source: PLoS One. 2022 Jan 24;17(1):e0262394. doi: 10.1371/journal.pone.0262394 (PMC8786187; doi:10.1371/journal.pone.0262394)
Supplement: S6 Table — (DOCX) [file pone.0262394.s006.docx]

**Table 6.** Conditional indirect effects of transformational leadership on safety compliance at values of perceived safety climate

| Moderator | Effect | Moderated  mediation effect | 95% CI of  moderated mediation effect |
| --- | --- | --- | --- |
| Path 1: TL→ FOL → SC | | | |
| Low felt safety climate (−1 SD) | .26^**^ | .07^*^ | [.012, .163] |
| High felt safety climate (+1 SD) |  | .16^**^ | [.090, .255] |
| Differ |  | .09^*^ | [.004, .189] |
| Path 2: TL→ SRT → SC | | | |
| Low felt safety climate (−1 SD) | −.120* | −.05^*^ | [−.108, −.004] |
| High felt safety climate (+1 SD) |  | . 02 | [−.004, .065] |
| Differ |  | .07^*^ | [.005, .149] |

*Note. N* = 309. ^*^ *p* < .05, ^**^ *p* < .01. TL is for transformational leadership. FOL is for felt obligation to leader, SRT is for safety risk tolerance. SC is for safety compliance,
