# Supplementary material for: Divergent effects of transformational leadership on safety compliance: A dual-path moderated mediation model
Source: PLoS One. 2022 Jan 24;17(1):e0262394. doi: 10.1371/journal.pone.0262394 (PMC8786187; doi:10.1371/journal.pone.0262394)
Supplement: S1 Questionnaire — (DOCX) [file pone.0262394.s007.docx]

**Questionnaire Items (English Version)**

**Transformational leadership**

1. My supervisor goes beyond self-interest for the good of the group
2. My supervisor instills pride in me
3. My supervisor acts in a way that builds my respect
4. My supervisor displays a sense of power and confidence.
5. My supervisor talks about the most important values and beliefs with me
6. My supervisor specifies the importance of having a strong sense of purpose
7. My supervisor considers the moral and ethical consequences of decisions
8. My supervisor emphasizes the importance of having a collective sense of mission
9. My supervisor talks optimistically about the future to me
10. My supervisor talks enthusiastically about what needs to be accomplished
11. My supervisor articulates a compelling vision of the future
12. My supervisor expresses confidence that goals will be achieved
13. My supervisor re-examines critical assumptions for appropriateness
14. My supervisor seeks differing perspectives from me when solving problems
15. My supervisor gets me look at problems from many different angles
16. My supervisor suggests new ways of looking at how to complete assignments
17. My supervisor spends time teaching and coaching me
18. My supervisor treats others as an individual rather than just as a member of a group
19. My supervisor considers me as having different needs, abilities, and aspirations from others
20. My supervisor helps me to develop their strength

**Felt obligation to leader**

1. I owe it to my leader to do whatever I can to come up with ideas/solutions to achieve his or her goal
2. I have an obligation to my leader to voice out my own opinions
3. I feel a personal obligation to produce constructive suggestions to help my leader achieve his or her goals
4. I owe it to my leader to do what I can to come up with brilliant ideas, to ensure that our customers are well served and satisfied
5. I would feel an obligation to take time from my personal schedule to solutions for my leader if he or she is needed

**Safety risk tolerance**

1. I can accept that a worker does not wear safety helmet when he is working in the second ﬂoor
2. I can accept that there is no safety sign or safety net around the reserved elevator hole
3. I can accept that there is inadequate personal protective equipment.
4. I can accept that safety nets do not cover the building when construction is in progress
5. I can accept that when excavation work is ongoing, the soils are placed at the edge of the foundation pit

Deleted three items

1. I can accept that a worker steps to an open-sided ﬂoor
2. I can accept that improper guards in place on power tool management resulted in easy body contact with power cables
3. I can accept that the crane coordination course does not include real training

**Safety climate**

1. Management places a strong emphasis on workplace health and safety
2. Safety is given a high priority by management
3. Management considers safety to be important

**Safety compliance**

1. I use all the necessary safety equipment to do my job
2. I use the correct safety procedures for carrying out my job
3. I ensure the highest levels of safety when I carry out my job

**问卷题项 (中文版本)**

**变革型领导**

1. 我的直接上级不图私利
2. 我的直接上级注重培养我的自豪感
3. 我的直接上级能获得我的尊重
4. 我的直接上级具有强大的气场和自信
5. 我的直接上级会与我讨论最重要的价值观和信仰
6. 我的直接上级明确指出拥有强烈目标感的重要性
7. 我的直接上级在决策时候会考虑道德和伦理后果
8. 我的直接上级强调拥有集体使命感的重要性
9. 我的直接上级会和我乐观地谈及未来
10. 我的直接上级会热情地谈论需要去做的事情
11. 我的直接上级会对我描绘出具有感染力的愿景
12. 我的直接上级对目标的完成展现出高度的自信
13. 我的直接上级会经常反思关键做法的正当性
14. 我的直接上级会从我这里寻求不同的观点以解决问题
15. 我的直接上级会让我从各种不同的角度来看待问题
16. 我的直接上级会为如何完成任务提出新的看法
17. 我的直接上级会花时间对我进行辅导
18. 我的直接上级不仅仅将我视为一个团队成员
19. 我的直接上级会关注我和其他人不同的需求、能力和追求
20. 我的直接上级帮助我提高自己

**感知到的对领导者义务**

1. 为了实现我直接领导的目标，我觉得自己有义务尽自己所能地出谋划策/解决问题
2. 为了实现我直接领导的目标，我觉得自己有义务向他（她）发表我的意见
3. 为了实现我直接领导的目标，我觉得自己有义务向他/她提出建设性的建议
4. 为了确保我们的客户得到良好的服务，我觉得自己要尽可能地向我的直接领导提出好的想法
5. 如果我的直接领导有需要，我觉得自己有义务从个人日程安排中抽出时间为他（她）解决问题

**安全风险容忍度**

1. 我可以接受一名工人在工作时可以没有戴安全帽
2. 我可以接受施工时安全网可以不覆盖建筑物
3. 我可以接受工地上个人防护设备是不足的
4. 我可以接受挖掘工作时挖出的土壤被放置在基坑的边缘
5. 我可以接受预留的电梯孔周围没有安全标志（安全网）

删除的三道题目

1. 我可以接受一名工人可以走到一个开放的楼层
2. 我可以接受电动工具管理的防护装置不到位，即使这会导致身体容易与电线接触
3. 我可以接受起重机协调课程不包括真正的培训

**安全氛围**

1. 管理层非常重视工作场所的健康和安全
2. 管理层对安全问题给予高度重视
3. 管理层认为安全是重要的

**安全遵守**

1. 我使用所有必要的安全设备来完成我的工作
2. 我使用正确的安全程序来执行我的工作
3. 我在执行工作时确保最高水平的安全
