# Supplementary material for: Divergent effects of transformational leadership on safety compliance: A dual-path moderated mediation model
Source: PLoS One. 2022 Jan 24;17(1):e0262394. doi: 10.1371/journal.pone.0262394 (PMC8786187; doi:10.1371/journal.pone.0262394)
Supplement: Attachment — Submitted filename: Response to Reviewers.docx [file pone.0262394.s009.docx]

It is a great honor for us that the reviewers provided these valuable comments. The comments indeed inspire us to improve the quality of our research essentially. We carefully studied them and tried our best to make a substantive revision or provide a detailed explanation. The specific revisions we made in response to reviewers’ comments are listed as follows.

**General Comments to the Author**

-------------------------------------------------------------------------------------------------------

**1. Is the manuscript technically sound, and do the data support the conclusions?**

Reviewer #1: Yes

Reviewer #2: Yes

**2. Has the statistical analysis been performed appropriately and rigorously?**

Reviewer #1: Yes

Reviewer #2: Yes

**3. Have the authors made all data underlying the findings in their manuscript fully** available?

Reviewer #1: Yes

Reviewer #2: Yes

**4. Is the manuscript presented in an intelligible fashion and written in standard English?**

Reviewer #1: Yes

Reviewer #2: Yes

**Responses**

We all authors would like to thank the two reviewers for their recognition of this article and their insightful comments. Next, we will respond to both authors' specific comments in detail on a one-to-one basis.

-------------------------------------------------------------------------------------------------------

**Reviewer #1**

-------------------------------------------------------------------------------------------------------

**Comment 1:** It is good to include workplace injuries in Chinese context and workplace injuries in Chinese construction in the introduction part.

**Responses:** We agree with the reviewer that it is necessary to include workplace injuries in the Chinese context and workplace injuries in Chinese construction in the introduction part because our study was conducted in the Chinese context. While we found the number of severe safety accidents and corresponding fatalities in the construction industry in China in 2018, we could not find the general data on nonfatal occupational injuries and illnesses released by the government or the media. As a supplement, we have added some examples to highlight the importance of safety management in the Chinese context. The specific modifications are as follows (also see page 2-3).

Workplace safety is a pressing concern for both researchers and practitioners in the field of safety management. Work-related injuries result in employee suffering and high organizational costs [1–3]. According to the U.S. Bureau of Labor Statistics survey, approximately 2.7 million nonfatal occupational injuries and illnesses were reported by private industry employers in the United States in 2020 [4]. In China, although the general data on similar workplace safety incidents are not publicly available; nonetheless, an increasing number of safety accidents—especially those that occur in the construction industry—are reported by the government and the media. For instance, a report from the Ministry of Emergency Management described the collapse of a plant that occurred on May 16, 2019 in Changning district, Shanghai and caused 12 deaths, 10 serious injuries, three minor injuries, and direct economic losses of approximately 34.3 million yuan [5]. As highlighted by Jiang and Xu (2020), safety accidents in the construction industry in China occur frequently and continue to increase, with 734 accidents and 840 fatalities reported in 2018 alone [6].

-------------------------------------------------------------------------------------------------------

**Comment 2:** Consistency is important. Safety performance/safety behavior and safety compliance cannot be used interchangeably (see Neal et al., 2000; <https://psycnet.apa.org/doi/10.1037/1076-8998.5.3.347>). The literature provided supports for the relationship between transformational leadership and safety performance/safety behaviors. The literature should focus on the relationship between transformational leadership and safety compliance. This is important because safety performance/behavior has two dimensions: (1) safety compliance and (2) safety involvement (see Neal et al., 2000; <https://psycnet.apa.org/doi/10.1037/1076-8998.5.3.347>). Should authors include findings on safety compliance, not safety performance to support the research problem.

**Responses:** We fully agree with the reviewer that safety performance and safety compliance cannot be used interchangeably in our study. Previously, scholars have found that the two safety-related behaviours - safety compliance and safety participation– may have different antecedent variables and formation processes. Therefore, we reorganized the literature and focused our discourse strictly on transformational leadership and safety compliance rather than transformational leadership and safety outcomes in general. The specific modifications are as follows (also see page 3-4).

Given the importance of workplace safety, a growing body of research has investigated the antecedents of safety compliance (i.e., a form of in-role safety performance that refers to mandated safety behaviors that are usually part of the formal requirements of employees’ work roles) [7], such as employee personality [e.g., 8,9], safety climate [e.g., 10,11], and supervisor helping behavior [e.g., 12]. Transformational leadership is another significant antecedent of safety compliance that has been widely explored in research on workplace safety in recent years [e.g., 8,13–16]. However, the body of research in this area has yielded equivocal results on the relationship between transformational leadership and safety compliance. Most studies have documented a positive impact of transformational leadership on safety compliance [e.g., 8,13,14]. However, some studies have found a non-significant association between these two variables [e.g., 15,17]. In a study by Inness et al. (2010) across different job sectors, employees’ perceptions of transformational leadership were found not related to their safety compliance in either primary or secondary jobs [15]. The researchers suggested that higher levels of transformational leadership may indirectly give employees more freedom in deciding whether to abide by existing organizational policies such as safety procedures, which conversely lead to variability in safety compliance.

Moreover, some studies in the field of safety management have even suggested that transformational leadership may negatively affect safety compliance. In viewing transformational leadership as a complex and multidimensional construct, Hoffmeister et al. (2014) examined the distinct effects of different dimensions of transformational leadership on safety compliance, and found one of these—individualized consideration—to be negatively related to safety compliance [16]. A similar relationship was reported in a recent study conducted by Xue, Fan, and Xie (2020), who found that senior managers’ safety inspiration (another dimension of transformational leadership) is negatively associated with safety compliance [18]. While these mixed findings have attracted substantial research interest, the relationship between transformational leadership and safety compliance remains unclear. Clarke (2013) argued that the mixed findings in the literature may be due to past studies’ failure to account for the negative role of transformational leadership and the complicated process through which transformational leadership influences safety compliance [19].

-------------------------------------------------------------------------------------------------------

**Comment 3:** Abbreviation Incorrect abbreviation: self-regulatory focus (SRF) theory instead of SFR.

**Responses:** Thanks to the reviewer for pointing out this mistake. We will be more rigorous in spelling in our future academic writing to avoid similar mistakes. Incorrect abbreviations of self-regulatory focus in two places have been corrected. The specific modifications are as follows (also see pages 4, 7).

To address this research gap, we use leader-member exchange (LMX) theory and self-regulatory focus (SRF) theory to explore the divergent effects of transformational leadership on safety compliance (Page 4).

SRF theory, proposed by Higgins (1998) [24], describes two distinct self-regulatory states that are involved in individual decision-making: promotion focus and prevention focus (Page 7).

-------------------------------------------------------------------------------------------------------

**Comment 4:** Lines 119-127: does unsafe behavior mean less or no safety compliance by employees? Needs more literature to support.

**Responses:** Thanks to the reviewer for raising this question, which prompted us to think further about the difference between unsafe behaviors and less safe compliance. While they are all risk-taking behaviors in the field of safety management, less safety compliance does not necessarily imply more unsafe behaviors. Therefore, we replaced the term “unsafe behavior” with the more accurate term“less safety compliance” and added more literature to support the relationship between safety risk tolerance and safety compliance as suggested by the reviewer. The specific modifications are as follows (also see page 8).

There is generally a lack of research investigating safety risk tolerance and the subsequent safety decisions regarding the safety behaviors [35,36]. Nonetheless, a growing body of primary and secondary evidence suggests that employees with higher safety risk tolerance are more likely to demonstrate risk-taking behaviors, such as reduced safety compliance. According to Bhandari and Hallowell (2022), employees with higher safety risk tolerance underestimate the safety risks in their work routines or simply regard safety activities as being “part of the job.” Such normalized risk-taking attitudes cause workers to resent the safety measures imposed by management, ultimately resulting in compliance issues such as the inappropriate use of personal protective equipment [37]. Given the dynamic nature of construction work, safety-related behaviors are difficult to monitor, regulate, and control in real-time. Globally, the construction industry has struggled to reduce workers’ willingness to accept risks (i.e., to lower their safety risk tolerance) through various safety training programs [38,39]. This serves to demonstrate the universal importance of safety risk tolerance in management practices on construction sites to prevent safety incidents. SRF theory holds that employees whose prevention focus is restrained and whose promotion focus is stimulated are more likely to be intrinsically motivated, overestimate their ability to control or prevent accidents, and ignore external threats [40]. This behavior leads to reduced risk identification, biased risk analysis, and inaccurate evaluations of the risks pertaining to safety accidents or injuries.

-------------------------------------------------------------------------------------------------------

**Comment 5:** Previous literature has found a direct relationship between transformational leadership and safety compliance. Why is this relationship not considered in this study? The following are examples of the relationship between transformational leadership and safety compliance:

(1) https://doi.org/10.1061/(ASCE)CO.1943-7862.0002023
(2) https://doi.org/10.1080/14330237.2020.1716551
(3) https://doi.org/10.1037/a0019380
(4) http://dx.doi.org/10.1016/j.ssci.2013.07.004

**Responses:** This is a great question. As the reviewer noted, most previous studies have found a positive and direct influence of transformational leadership on safety compliance. In our study, if we simply look at the relationship between transformational leadership and safety compliance without introducing the two mediating mechanisms, we can get the same conclusion (see Model 2 in Table 4, page 21).

However, the starting point of our research challenges the premise that the influence of transformational leadership on safety compliance is necessarily positive. In some of the previous studies, although not many, we have found that the relationship between transformational leadership and safety compliance is likely to be insignificant or even negative. For example, in Kark and Katz-Navon’s (2015) study, we can find a non-significant correlation between transformational leadership and safety compliance, though the two researchers do not directly propose a hypothesis for the relationship between these two variables. In the third article recommended by the reviewer (https://doi.org/10.1037/a0019380), we find direct evidence of this. In that article, Inness et al. (2010) found that transformational leadership is not related to safety compliance in either the primary or secondary jobs. The researchers suggested that higher levels of transformational leadership may indirectly give employees greater latitude to use their discretion in deciding whether to comply with existing organizational policies such as safety procedures, which results in variability in individual safety compliance.

In general, the direct relationship between transformational leadership and safety compliance is not the purpose of our study. We aim to explain the different influence processes that transformational leadership may have on safety compliance by introducing the two different mechanisms. By doing so, we provide new insights into the equivocal results among transformational leadership and safety compliance in the field of safety management. We are very grateful to the reviewers for their generosity in providing these four relevant articles, which were essential to refine the ideas in our paper. Therefore, we have cited all of them in our revision. Thank you.

-------------------------------------------------------------------------------------------------------

**Comment 6:** Please explain the approach to data analysis before the Results section.

**Responses:** We fully agree with the reviewer that the approach to data analysis is vital for our research. It allows the reading to be more explicit about how our research results were obtained. The specific modifications are as follows (also see page 13-14).

**Method of Analysis**

In this study, we adopted software SPSS 25.0, SPSS AMOS, and MPLUS 7.0 as data analysis tools to analyze the 309 valid data. The data analysis approaches involved in this research include (1) confirmatory factor analysis (CFA), (2) tests of convergent and discriminant validity, (3) descriptive statistics and correlations, and (4) hypotheses testing.

We ran a CFA using SPSS AMOS to check whether the hypothesized five-factor model matched the actual data. We used five general indexes to evaluate the model ﬁt: CFI, IFI, TLI, RMSEA, and SRMR. Additionally, we conducted tests of convergent and discriminant validity for the hypothesized variables. Specifically, we used the indicators of average variance extracted (AVE) and composite reliability (CR) to verify the convergent validity and the comparisons of AVE with the squared correlations involving the variables (AVE/*r^2^*) to verify the discriminant validity [58].

While testing the direct effects among variables (Hypotheses 1a, 1b, 2a, and 2b), we conducted hierarchical linear regression in SPSS 25.0. In the process of analysis, Model 1 includes the control variables and the dependent variable of safety compliance; Model 2 includes the control variables, the independent variable of transformational leadership, and the dependent variable of safety compliance; Model 3 includes the control variables, the independent variable of transformational leadership, and the dependent variable of felt obligation to leader; Model 4 includes the control variables, the independent variable of transformational leadership, and the dependent variable of safety risk tolerance; Model 5 includes the control variables, the independent variable of felt obligation to leader, and the dependent variable of safety compliance; Model 6 includes the control variables, the independent variable of safety risk tolerance, and the dependent variable of safety compliance.

To test the hypothesized mediation effects, we used a bootstrap method, applying MPLUS 7.0. We used 5000-sample bootstrapping to yield 95% bias-corrected conﬁdence intervals. If the conﬁdence interval excludes zero, it leads to the inference that the mediation eﬀect is significant. In the final step, we then used MPLUS 7.0 to quantify the difference in mediation effects between low (-1SD) and high (+1SD) levels of perceived safety climate. If the conﬁdence interval of the difference excludes zero, it leads to the inference that the moderating eﬀect is significant.

-------------------------------------------------------------------------------------------------------

**Comment 7:** (1) Average variance extraction (AVE) and factor loadings are not reported. These tests are important to measure a construct - convergent validity. (2) Discriminant validity should be tested to ensure the distinct between constructs (AVE > r2).

**Responses:** We highly agree with the reviewer that the convergent and discriminant validity should be calculated and reported in our research. According to Ahmad et al. (2016), the convergent validity can be evaluated by indicators of AVE (average variance extracted) and CR (composite reliability), while the discriminant validity can be assessed by the indicator of AVE/*r^2^* (the comparisons of AVE with the squared correlations involving the variables). The specific modifications are as follows (also see page 17).

**Tests of convergent and discriminant validity**

According to Ahmad et al. (2016), AVE and CR can be used to assess the convergent validity, while AVE/*r^2^* can be used to test the discriminant validity [58]. As shown in Table 2, all the CR values are above 0.70, and the AVE values are above 0.5, indicating good convergent validity for all variables. Moreover, the results of discriminant validity tests (AVE/*r^2^* > 1) for all variables suggest that the amount of the variance capture by each variable is greater than the shared variance with the other variables, indicating that the variables are distinct from one another.

**Table 2.** Tests for Convergent and Discriminant Validity

| **Variables** | **Convergent validity** | **Discriminant validity** |
| --- | --- | --- |
| Transformational leadership |  |  |
| CR | 0.906 | AVE/*r^2^* > 1 |
| AVE | 0.706 |  |
| Felt obligation to leader |  |  |
| CR | 0.883 | AVE/*r^2^* > 1 |
| AVE | 0.604 |  |
| Safety risk tolerance |  |  |
| CR | 0.825 | AVE/*r*^2^ > 1 |
| AVE | 0.556 |  |
| Perceived safety climate, |  |  |
| CR | 0.885 | AVE/*r^2^* > 1 |
| AVE | 0.721 |  |
| Safety compliance |  |  |
| CR | 0.864 | AVE/*r^2^* > 1 |
| AVE | 0.697 |  |

*Ahmad S, Zulkurnain N, Khairushalimi F. Assessing the Validity and Reliability of a Measurement Model in Structural Equation Modeling (SEM). BJMCS. 2016;15: 1–8.*

-------------------------------------------------------------------------------------------------------

**Comment 8:** It is good to report the R2 to validate the gaps and conflicting results in safety performance research, especially on safety compliance. The report on R2 could support for theoretical and practical contributions section.

**Responses:** Thanks to the reviewer for this advice. Following a similar approach in the relevant literature (Ugwu et al., 2020) recommended by the reviewer, we re-performed the data using the hierarchical linear regression in SPSS software. The R-square has been calculated in Table 4 and reported in the result section. As indicated by the reviewer and the results we obtained, most of the variance in safety compliance is captured by the positive influence process of transformational leadership and less by the negative influence process of transformational leadership. This result validates our arguments about the divergent effects of transformational leadership on safety compliance to some extent. The specific modifications are as follows (also see page 20).

**Hypothesis Testing**

Table 4 presents the results of hierarchical regression analyses for the Hypotheses 1a, 1b, 2a, and 2b. As predicted, controlling for the above demographic variables, we found that transformational leadership is positively related to felt obligation to leaders (β =.26, p <.01, ΔR² = 0.03), and transformational leadership accounted for 3% of the variance in felt obligation to leaders. Similarly, we found that transformational leadership is positively related to safety risk tolerance (β =.22, p <.01, ΔR² = 0.04), and transformational leadership accounted for 4% of the variance in safety risk tolerance. We also found that felt obligation to leaders is positively related to safety compliance (β =.27, p <.01, ΔR² = 0.12), whereas safety risk tolerance is negatively related to safety compliance (β = −.11, p <.05, ΔR² = 0.01), suggesting that felt obligation to leaders and safety risk tolerance accounted for 12% and 1% of the variance of safety compliance, respectively. Overall, these results support Hypotheses 1a, 1b, 2a, and 2b.

*Ugwu FO, Idike AN, Ibiam OE, Akwara FA, Okorie CO. Transformational leadership and management safety practices: Their role in the relationship between work pressure and compliance with safety work behaviour in a health-care sector industry. Journal of Psychology in Africa. 2020;30: 1–8. doi:10.1080/14330237.2020.1716551*

-------------------------------------------------------------------------------------------------------

**Comment 9:** A proofreading is strongly recommended to improve the quality of the manuscript.

**Responses:** Many thanks to the reviewer for the last advice. We proofread the manuscript in three aspects to further improve the quality of our manuscript. First, the two of the authors, who are relatively good at English, once again checked the wording and grammar of the paper thoroughly. Second, we double-checked the entire manuscript with Grammarly, a specialized writing assistant ([www.grammarly.com](http://www.grammarly.com/)), to ensure that our writing is as clear and mistake-free as possible. Third, we sent the revised article to AsiaEdit, a leading academic editing agency ([https://asiaedit.com](https://asiaedit.com/)), for the final proofreading and revisions. We believe that the writing quality in this version has been greatly improved. We thank the reviewer for this advice.

-------------------------------------------------------------------------------------------------------

**Reviewer #2**

-------------------------------------------------------------------------------------------------------

**Comment:** This study investigates the relationship between transformational leadership and safety performance. The study attempts to provide empirical understating on the divergent influence of transformational leadership on safety compliance. Very interesting study! Overall, the design enables for causal inferences, statistics, analyses are appropriate/ sufficient, and conclusions are evidence-based.

**Responses:** Thank you to the reviewer for the overall recognition of our paper. Next, we will answer or revise it to address the specific issues raised by the reviewer.

-------------------------------------------------------------------------------------------------------

**Comment 1:** The specific items for all the measures used should be included as-is (e.g., as Supplemental Information). For instance, it is unclear which three items were discarded in the ‘Safety risk tolerance’ measures. The raw data provided do not include labels of the individual questions. The English and Chinese versions should be made available.

**Responses:** This is good advice. We have attached the questionnaire items for all the measures both in English and Chinese to the Supplemental Information. Also, by following the comments of the reviewer, we marked the deleted question items in the ‘safety risk tolerance’ measures (e.g., I can accept that a worker steps to an open-sided ﬂoor) in the “S1_Questionnaire Items (English and Chinese Versions)” and included labels of the individual questions in the raw data we provided. We now believe the readers will have a clearer understanding of how each variable is measured. Thanks again to the reviewer for this advice.

-------------------------------------------------------------------------------------------------------

**Comment 2:** In addition, the procedure of standard translation-back-translation should be elaborated further to offer explicit justification of the validity of translated measures.

**Responses:** Thank you for raising this comment. We have detailed how we conducted the procedure of standard translation and back-translation. By doing so, we can now provide further justification for the validity of translated measures. The specific modifications are as follows (also see page 11-12). Thank you :)

**Measurement**

All the above measures were administered in Chinese and translated from English into Chinese by using a procedure of standard translation and back-translation to ensure validity [52]. At the initial translation stage, two experts with a high level of English, one being the author of our research and the other being a manager from the construction industry, translated all the questionnaire items from English to Chinese. They then proceeded to check, discuss, and modify the translated Chinese questionnaire items until both agreed on the results of all the items. After that, in the back-translation stage, we paid an English graduate student to re-translate the Chinese questionnaire items obtained in the previous stage into English and compare them with the original English questionnaire items. If the meanings expressed do not match, the translation phase is repeated until they agree on the meaning.

-------------------------------------------------------------------------------------------------------

**Comment 3:** The authors use 20 items from MLQ as a measure of transformational leadership. They reference an old 1999 study as a justification of this decision. Whilst MLQ is widely used, there is a recent criticism on the validity of its constructs (e.g., “Measuring leadership an assessment of the Multifactor Leadership Questionnaire”, https://doi.org/10.1371/journal.pone.0254329)

**Responses:** That is an excellent question that we had not noticed before. Our study simply borrowed or adapted scales developed by domain researchers without considering the possible criticism on them. We really learned a lot by reading the study referred by the reviewer.

As pointed out by the reviewer, the literature we used to justify the scale of transformational leadership is relatively old. However, this literature one of the most classic references on the measurement of transformational leadership. In Google Scholar, this literature has over 5,500 citations, many of which are from recent articles published in high quality journals. For example. Kark et al. (2015) developed two scenarios drawing on items from the Multifactor Leadership Questionnaire (MLQ) to test the relationships among transformational leadership, promotion situational focus, and safety initiative (in Journal of Applied Psychology). A recent systematic review of transformational leadership theory (in The Leadership Quarterly) suggested that more than 86% of empirical studies utilized a single measure of transformational leaders (the MLQ) in 2004, while the number has decreased year-over-year to 68% by 2019. However, a further analysis of trends in operationalizations of transformational leadership warrants that even when researchers use measures other than the MLQ, they often convert non-MLQ measures into the dimensional labels used by the MLQ or suggest scale equivalence (Siangchokyoo et al., 2020), implying that the scale of transformational leadership in MLQ developed by Avolio, Bass, and Jung (1999) is still widely recognized by the academic community.

While MLQ is widely used, we agree with the reviewer that the use of transformational leadership scale from MLQ is not flawless. A recent debate on MLQ has focused on its specification chosen——whether the structure of transformational leadership is a latent or aggregated model. According to Batista-Foguet, Esteve, and van Witteloostuijn (2021), the factorial structure of transformational leadership in MLQ should be classified formative (a latent model), rather than reflective (an aggregated model). The model misspecification of transformational leadership may result in erroneous parameter estimates and misleading statistical tests.

Although the three authors also note that the generalizability of their findings are limited owing to the distinct characteristics of the study sample (police force data within a very hierarchical organization), we recognize one of their key ideas that researchers may take a dimensional approach to understand the relationships between the specific dimensions of transformational leadership (e.g., inspirational motivation) and the various types of outcomes. This idea is supported to some extent when we elaborated the possible negative relationship between transformational leadership and safety compliance (see page 3-4, line 54-62). We are grateful to the reviewer for recommending the literature that offers us a good direction for future study; that is, researchers could open the "black box" of transformational leadership to explore the distinct pathways of specific dimensions of transformational leadership affecting safety compliance, and this will add explanations for why research on the relationship between transformational leadership and safety compliance has yielded equivocal results. Based on the reviewer’s comment and our reflections on it, we added an additional research limitation and future direction linked to transformational leadership. The specific modifications are as follows (also see page 28).

The third limitation of our study concerns the use of the transformational leadership scale in the MLQ. While MLQ is still widely recognized by the academic community, a recent criticism on the validity of its constructs suggested that the factorial structure of transformational leadership in MLQ should be classified formative (a latent model), rather than reflective (an aggregated model) [68]. The model misspecification of transformational leadership may result in erroneous parameter estimates and misleading statistical tests. This idea is supported to some extent when we elaborated on the possible negative relationship between transformational leadership and safety compliance. For instance, Hoffmeister et al. (2014) examined the distinct effects of different dimensions of transformational leadership on safety compliance and found one of these—individualized consideration—to be negatively related to safety compliance [16]. Therefore, future research could open the "black box" of transformational leadership to explore the distinct pathways of specific dimensions of transformational leadership affecting safety compliance. This will add explanations for the fundamental understanding of our research question: why has research on the relationship between transformational leadership and safety compliance yielded equivocal results?

-------------------------------------------------------------------------------------------------------

**Comment 4:** Why did the authors not randomize the items/ measures to minimize potential order effects (e.g., two or three versions of the same items/ measures, but in different order)?

**Responses:** We would like to thank the reviewer for raising this methodological issue. In psychological research, researchers usually pay more attention to the issue of common method bias. This was also the case in our study, so more effort was spent on a two-round questionnaire collection. Regarding the response-order effects raised by the reviewer, we consider it unlikely to have a significant impact on the estimation of our results. On the one hand, our surveys were conducted in two rounds, with each round taking only a short period of time to complete the survey. On the other hand, considering the education level and cognitive ability of construction workers, we guaranteed that special staff would be available to answer questions and solve problems when the construction workers were filling out the surveys. With these two proven methods, we might have significantly reduced the shortcut behaviors of the construction workers and thus have addressed the response order effects at the root (Krosnick and Alwin, 1987).

*Krosnick, J. A., & Alwin, D. F. (1987). An evaluation of a cognitive theory of response-order effects in survey measurement. Public opinion quarterly, 51(2), 201-219.*

-------------------------------------------------------------------------------------------------------

**Comment 5:** It is currently not crystal clear what the authors mean that they controlled for the demographical information variables (see line 226). I could just assume that that they refer to controlling for demographical variables within the proposed research method (as seen in line 258). If this is what they mean, then this should be clarified earlier.

**Responses:** Yes. We referred to controlling for demographical variables within the proposed research method. Many thanks to the reviewer for raising this point, which is consistent with the first reviewer's comment that the approach to data analysis should be clarified in advance. We apologize for not clarifying this point earlier and for confusing your reading. We have detailed the use of control variables in the methods section following the two reviewers’ comments. The specific modifications are as follows (also see page 14 and 19).

While testing the direct effects among variables (Hypotheses 1a, 1b, 2a, and 2b), we conducted hierarchical linear regression in SPSS 25.0. In the process of analysis, Model 1 includes the control variables and the dependent variable of safety compliance; Model 2 includes the control variables, the independent variable of transformational leadership, and the dependent variable of safety compliance; Model 3 includes the control variables, the independent variable of transformational leadership, and the dependent variable of felt obligation to leader; Model 4 includes the control variables, the independent variable of transformational leadership, and the dependent variable of safety risk tolerance; Model 5 includes the control variables, the independent variable of felt obligation to leader, and the dependent variable of safety compliance; Model 6 includes the control variables, the independent variable of safety risk tolerance, and the dependent variable of safety compliance.

**Table 3**. Descriptive statistics, reliabilities, and inter-correlations among the hypothesized variables

| **Variables** | **M** | **SD** | **1** | **2** | **3** | **4** | **5** | **6** | **7** | **8** |
| --- | --- | --- | --- | --- | --- | --- | --- | --- | --- | --- |
| 1. Age | 39.18 | 8.80 | — |  |  |  |  |  |  |  |
| 2. Gender | 1.12 | .33 | −.07 | — |  |  |  |  |  |  |
| 3. Education | 1.87 | .63 | −.29^**^ | .01 | — |  |  |  |  |  |
| 4. Transformational leadership | 3.85 | .66 | −.20^**^ | .00 | .08 | (.94) |  |  |  |  |
| 5. Felt obligation to leader | 3.36 | 1.03 | −.09 | .09 | .05 | .18^**^ | (.89) |  |  |  |
| 6. Safety risk tolerance | 3.48 | .81 | .01 | −.05 | .12^*^ | .18^**^ | .00 | (.82) |  |  |
| 7. Felt safety climate | 5.42 | 1.21 | −.11 | −.05 | .13^*^ | .45^**^ | −.11 | .21^**^ | (.86) |  |
| 8. Safety compliance | 4.08 | .79 | .01 | .08 | −.06 | .12^*^ | .34^**^ | −.12^*^ | −.03 | (.88) |

*Note: N*=309. ^*^ *p* < .05, ^**^ *p* < .01. Internal consistency reliabilities are reported in parentheses along diagonal.

-------------------------------------------------------------------------------------------------------

**Comment 6:** The term ‘safety performance’ should be clarified upfront in the introduction (currently it is explained in line 82 but not in the intro).

**Responses:** This is a good suggestion. Combined with the first reviewer's second comment that the authors should include findings on safety compliance instead of safety performance, we have focused our discourse strictly on transformational leadership and safety compliance and have defined the term "safety compliance" when it first appears in the introduction. The specific modifications are as follows (also see page 3).

Given the importance of workplace safety, a growing body of research has investigated the antecedents of safety compliance (i.e., a form of in-role safety performance that refers to mandated safety behaviors that are usually part of the formal requirements of employees’ work roles) [7], such as employee personality [e.g., 8,9], safety climate [e.g., 10,11], and supervisor helping behavior [e.g., 12].

-------------------------------------------------------------------------------------------------------

**Comment 7:** There was an in-text citation in line 25 that is not available in the reference list.

**Responses:** We thank the reviewers for noting this. We have updated the data on nonfatal workplace injuries in the United States based on the latest survey from the Bureau of Labor Statistics (BLS), with sources attached. The specific modifications are as follows (also see page 2).

According to the U.S. Bureau of Labor Statistics survey, approximately 2.7 million nonfatal occupational injuries and illnesses were reported by private industry employers in the United States in 2020 [4].

*Bureau of Labor Statistics (BLS). Employer-reported workplace injuries and illnesses – 2020. In: Survey of Occupational Injuries and Illnesses, [Internet]. 3 Nov 2021 [cited 1 Dec 2021]. Available: https://webcache.googleusercontent.com/search?q=cache:_KDgstNFn5AJ:https://www.bls.gov/news.release/pdf/osh.pdf+&cd=1&hl=zh-CN&ct=clnk&gl=hk*

-------------------------------------------------------------------------------------------------------
