# Supplementary material for: Divergent effects of transformational leadership on safety compliance: A dual-path moderated mediation model
Source: PLoS One. 2022 Jan 24;17(1):e0262394. doi: 10.1371/journal.pone.0262394 (PMC8786187; doi:10.1371/journal.pone.0262394)
Supplement: Peer review history

# Peer review: Decision Letter 0

**DOI:** 10.1371/journal.pone.0262394.r001

**Authors:** Abroon Qazi

21 Oct 2021

PONE-D-21-18005Divergent Effects of Transformational Leadership on Safety Compliance: A Dual-Path Moderated Mediation ModelPLOS ONE

Dear Dr. Zheng,

Thank you for submitting your manuscript to PLOS ONE. After careful consideration, we feel that it has merit but does not fully meet PLOS ONE’s publication criteria as it currently stands. Therefore, we invite you to submit a revised version of the manuscript that addresses the points raised during the review process.

Please submit your revised manuscript by Dec 05 2021 11:59PM. If you will need more time than this to complete your revisions, please reply to this message or contact the journal office at plosone@plos.org. When you're ready to submit your revision, log on to https://www.editorialmanager.com/pone/ and select the 'Submissions Needing Revision' folder to locate your manuscript file.

Please include the following items when submitting your revised manuscript:

- A rebuttal letter that responds to each point raised by the academic editor and reviewer(s). You should upload this letter as a separate file labeled 'Response to Reviewers'.
- A marked-up copy of your manuscript that highlights changes made to the original version. You should upload this as a separate file labeled 'Revised Manuscript with Track Changes'.
- An unmarked version of your revised paper without tracked changes. You should upload this as a separate file labeled 'Manuscript'.

If you would like to make changes to your financial disclosure, please include your updated statement in your cover letter. Guidelines for resubmitting your figure files are available below the reviewer comments at the end of this letter.

If applicable, we recommend that you deposit your laboratory protocols in protocols.io to enhance the reproducibility of your results. Protocols.io assigns your protocol its own identifier (DOI) so that it can be cited independently in the future. For instructions see: https://journals.plos.org/plosone/s/submission-guidelines#loc-laboratory-protocols. Additionally, PLOS ONE offers an option for publishing peer-reviewed Lab Protocol articles, which describe protocols hosted on protocols.io. Read more information on sharing protocols at https://plos.org/protocols?utm_medium=editorial-email&utm_source=authorletters&utm_campaign=protocols.

We look forward to receiving your revised manuscript.

Kind regards,

Abroon Qazi, Ph.D.

Academic Editor

PLOS ONE

Journal Requirements:

When submitting your revision, we need you to address these additional requirements.

1. Please ensure that your manuscript meets PLOS ONE's style requirements, including those for file naming. The PLOS ONE style templates can be found at

https://journals.plos.org/plosone/s/file?id=wjVg/PLOSOne_formatting_sample_main_body.pdf and https://journals.plos.org/plosone/s/file?id=ba62/PLOSOne_formatting_sample_title_authors_affiliations.pdf

Additional Editor Comments (if provided):

The two reviewers provide recommendations for a revision.

The authors should be invited to prepare a revision that addresses the suggestions of each of the reviewers.

A summary of the revisions should be provided, collated to the original reviewer and editor comments.

[Note: HTML markup is below. Please do not edit.]

Reviewers' comments:

Reviewer's Responses to Questions

**Comments to the Author**

1. Is the manuscript technically sound, and do the data support the conclusions?

The manuscript must describe a technically sound piece of scientific research with data that supports the conclusions. Experiments must have been conducted rigorously, with appropriate controls, replication, and sample sizes. The conclusions must be drawn appropriately based on the data presented. 

Reviewer #1: Yes

Reviewer #2: Yes

**********

2. Has the statistical analysis been performed appropriately and rigorously? 

Reviewer #1: Yes

Reviewer #2: Yes

**********

3. Have the authors made all data underlying the findings in their manuscript fully available?

The PLOS Data policy requires authors to make all data underlying the findings described in their manuscript fully available without restriction, with rare exception (please refer to the Data Availability Statement in the manuscript PDF file). The data should be provided as part of the manuscript or its supporting information, or deposited to a public repository. For example, in addition to summary statistics, the data points behind means, medians and variance measures should be available. If there are restrictions on publicly sharing data—e.g. participant privacy or use of data from a third party—those must be specified.

Reviewer #1: Yes

Reviewer #2: Yes

**********

4. Is the manuscript presented in an intelligible fashion and written in standard English?

PLOS ONE does not copyedit accepted manuscripts, so the language in submitted articles must be clear, correct, and unambiguous. Any typographical or grammatical errors should be corrected at revision, so please note any specific errors here.

Reviewer #1: Yes

Reviewer #2: Yes

**********

5. Review Comments to the Author

Please use the space provided to explain your answers to the questions above. You may also include additional comments for the author, including concerns about dual publication, research ethics, or publication ethics. (Please upload your review as an attachment if it exceeds 20,000 characters)

Reviewer #1: 1. It is good to include workplace injuries in Chinese context and workplace injuries in Chinese construction in the introduction part.

2. Consistency is important. Safety performance/safety behavior and safety compliance cannot be used interchangeably (see Neal et al., 2000; https://psycnet.apa.org/doi/10.1037/1076-8998.5.3.347).

3. The literature provided supports for the relationship between transformational leadership and safety performance/safety behaviors. The literature should focus on the relationship between transformational leadership and safety compliance. This is important because safety performance/behavior has two dimensions: (1) safety compliance and (2) safety involvement (see Neal et al., 2000; https://psycnet.apa.org/doi/10.1037/1076-8998.5.3.347).

4. Should Authors should include findings on safety compliance, not safety performance to support the research problem.

5. Abbreviation Incorrect abbreviation: self-regulatory focus (SRF) theory instead of SFR.

6. Lines 119-127: does unsafe behavior mean less or no safety compliance by employees? Needs more literature to support.

7. Previous literature has found a direct relationship between transformational leadership and safety compliance. Why is this relationship not considered in this study?

The following are examples of the relationship between transformational leadership and safety compliance:

https://doi.org/10.1061/(ASCE)CO.1943-7862.0002023 https://doi.org/10.1080/14330237.2020.1716551 https://doi.org/10.1037/a0019380 http://dx.doi.org/10.1016/j.ssci.2013.07.004

8. Please explain the approach to data analysis before the Results section

9. Average variance extraction (AVE) and factor loadings are not reported. These tests are important to measure a construct - convergent validity.

10. Discriminant validity should be tested to ensure the distinct between constructs (AVE > r2).

11. It is good to report the R2 to validate the gaps and conflicting results in safety performance research, especially on safety compliance. The report on R2 could support for theoretical and practical contributions section.

12. A proofreading is strongly recommended to improve the quality of the manuscript.

Reviewer #2: This study investigates the relationship between transformational leadership and safety performance. The study attempts to provide empirical understating on the divergent influence of transformational leadership on safety compliance. Very interesting study!

Overall, the design enables for causal inferences, statistics, analyses are appropriate/ sufficient, and conclusions are evidence-based.

There are several improvement areas that should be addressed:

The specific items for all the measures used should be included as-is (e.g., as Supplemental Information). For instance, it is unclear which three items were discarded in the ‘Safety risk tolerance’ measures. The raw data provided do not include labels of the individual questions.

The English and Chinese versions should be made available. In addition, the procedure of standard translation-back-translation should be elaborated further to offer explicit justification of the validity of translated measures.

The authors use 20 items from MLQ as a measure of transformational leadership. They reference an old 1999 study as a justification of this decision. Whilst MLQ is widely used, there is a recent criticism on the validity of its constructs (e.g., “Measuring leadership an assessment of the Multifactor Leadership Questionnaire”, https://doi.org/10.1371/journal.pone.0254329)

Why did the authors not randomize the items/ measures to minimize potential order effects (e.g., two or three versions of the same items/ measures, but in different order)?

It is currently not crystal clear what the authors mean that they controlled for the demographical information variables (see line 226). I could just assume that that they refer to controlling for demographical variables within the proposed research method (as seen in line 258). If this is what they mean, then this should be clarified earlier.

The term ‘safety performance’ should be clarified upfront in the introduction (currently it is explained in line 82 but not in the intro).

There was an in-text citation in line 25 that is not available in the reference list.

**********

6. PLOS authors have the option to publish the peer review history of their article (what does this mean?). If published, this will include your full peer review and any attached files.

If you choose “no”, your identity will remain anonymous but your review may still be made public.

**Do you want your identity to be public for this peer review?** For information about this choice, including consent withdrawal, please see our Privacy Policy.

Reviewer #1: No

Reviewer #2: No

[NOTE: If reviewer comments were submitted as an attachment file, they will be attached to this email and accessible via the submission site. Please log into your account, locate the manuscript record, and check for the action link "View Attachments". If this link does not appear, there are no attachment files.]

While revising your submission, please upload your figure files to the Preflight Analysis and Conversion Engine (PACE) digital diagnostic tool, https://pacev2.apexcovantage.com/. PACE helps ensure that figures meet PLOS requirements. To use PACE, you must first register as a user. Registration is free. Then, login and navigate to the UPLOAD tab, where you will find detailed instructions on how to use the tool. If you encounter any issues or have any questions when using PACE, please email PLOS at figures@plos.org. Please note that Supporting Information files do not need this step.

# Author comment: Author response to Decision Letter 0

**DOI:** 10.1371/journal.pone.0262394.r002

3 Dec 2021

It is a great honor for us that the reviewers provided these valuable comments. The comments indeed inspire us to improve the quality of our research essentially. We carefully studied them and tried our best to make a substantive revision or provide a detailed explanation. The specific revisions we made in response to reviewers’ comments have been attached to the submission system.

Attachment Submitted filename: Response to Reviewers.docx Click here for additional data file.

# Peer review: Decision Letter 1

**DOI:** 10.1371/journal.pone.0262394.r003

**Authors:** Abroon Qazi

23 Dec 2021

Divergent Effects of Transformational Leadership on Safety Compliance: A Dual-Path Moderated Mediation Model

PONE-D-21-18005R1

Dear Dr. Zheng,

We’re pleased to inform you that your manuscript has been judged scientifically suitable for publication and will be formally accepted for publication once it meets all outstanding technical requirements.

Within one week, you’ll receive an e-mail detailing the required amendments. When these have been addressed, you’ll receive a formal acceptance letter and your manuscript will be scheduled for publication.

An invoice for payment will follow shortly after the formal acceptance. To ensure an efficient process, please log into Editorial Manager at http://www.editorialmanager.com/pone/, click the 'Update My Information' link at the top of the page, and double check that your user information is up-to-date. If you have any billing related questions, please contact our Author Billing department directly at authorbilling@plos.org.

If your institution or institutions have a press office, please notify them about your upcoming paper to help maximize its impact. If they’ll be preparing press materials, please inform our press team as soon as possible -- no later than 48 hours after receiving the formal acceptance. Your manuscript will remain under strict press embargo until 2 pm Eastern Time on the date of publication. For more information, please contact onepress@plos.org.

Kind regards,

Abroon Qazi, Ph.D.

Academic Editor

PLOS ONE

Additional Editor Comments (optional):

Reviewers' comments:

Reviewer's Responses to Questions

**Comments to the Author**

1. If the authors have adequately addressed your comments raised in a previous round of review and you feel that this manuscript is now acceptable for publication, you may indicate that here to bypass the “Comments to the Author” section, enter your conflict of interest statement in the “Confidential to Editor” section, and submit your "Accept" recommendation.

Reviewer #2: All comments have been addressed

**********

2. Is the manuscript technically sound, and do the data support the conclusions?

The manuscript must describe a technically sound piece of scientific research with data that supports the conclusions. Experiments must have been conducted rigorously, with appropriate controls, replication, and sample sizes. The conclusions must be drawn appropriately based on the data presented. 

Reviewer #2: Yes

**********

3. Has the statistical analysis been performed appropriately and rigorously? 

Reviewer #2: Yes

**********

4. Have the authors made all data underlying the findings in their manuscript fully available?

The PLOS Data policy requires authors to make all data underlying the findings described in their manuscript fully available without restriction, with rare exception (please refer to the Data Availability Statement in the manuscript PDF file). The data should be provided as part of the manuscript or its supporting information, or deposited to a public repository. For example, in addition to summary statistics, the data points behind means, medians and variance measures should be available. If there are restrictions on publicly sharing data—e.g. participant privacy or use of data from a third party—those must be specified.

Reviewer #2: Yes

**********

5. Is the manuscript presented in an intelligible fashion and written in standard English?

PLOS ONE does not copyedit accepted manuscripts, so the language in submitted articles must be clear, correct, and unambiguous. Any typographical or grammatical errors should be corrected at revision, so please note any specific errors here.

Reviewer #2: Yes

**********

6. Review Comments to the Author

Please use the space provided to explain your answers to the questions above. You may also include additional comments for the author, including concerns about dual publication, research ethics, or publication ethics. (Please upload your review as an attachment if it exceeds 20,000 characters)

Reviewer #2: The authors have adequately addressed all comments.

Just two small notes:

For Response 2, I would include “fluency” when describing the level of English.

For Response 2, I would clarify about “English graduate” –Does it mean someone who is from the United Kingdom or someone who proficient in English?

**********

7. PLOS authors have the option to publish the peer review history of their article (what does this mean?). If published, this will include your full peer review and any attached files.

If you choose “no”, your identity will remain anonymous but your review may still be made public.

**Do you want your identity to be public for this peer review?** For information about this choice, including consent withdrawal, please see our Privacy Policy.

Reviewer #2: No

# Editor report: Acceptance letter

**DOI:** 10.1371/journal.pone.0262394.r004

**Authors:** Abroon Qazi

30 Dec 2021

PONE-D-21-18005R1 

Divergent Effects of Transformational Leadership on Safety Compliance: A Dual-Path Moderated Mediation Model 

Dear Dr. Zheng:

I'm pleased to inform you that your manuscript has been deemed suitable for publication in PLOS ONE. Congratulations! Your manuscript is now with our production department. 

If your institution or institutions have a press office, please let them know about your upcoming paper now to help maximize its impact. If they'll be preparing press materials, please inform our press team within the next 48 hours. Your manuscript will remain under strict press embargo until 2 pm Eastern Time on the date of publication. For more information please contact onepress@plos.org.

If we can help with anything else, please email us at plosone@plos.org. 

Thank you for submitting your work to PLOS ONE and supporting open access. 

Kind regards, 

PLOS ONE Editorial Office Staff

on behalf of

Dr. Abroon Qazi 

Academic Editor

PLOS ONE
